# Supplementary material for: Forecasting Diabetes Cases Prevented and Cost Savings Associated with Population Increases of Walking in the Greater Toronto and Hamilton Area, Canada
Source: Int J Environ Res Public Health. 2021 Jul 31;18(15):8127. doi: 10.3390/ijerph18158127 (PMC8345977; doi:10.3390/ijerph18158127)
Supplement: Supplementary file 1 [file ijerph-18-08127-s001.zip › ijerph-1285617-supplementary.pdf]

TableS1. Diabetes Population Risk Tool (DPoRT) Algorithm [1]

Males:

μ = 10.3062
− 0.3629 × hypertension
− 0.3483 × heart disease
− 0.5697 × non-white ethnicity
− 0.0585 × smoker
+ 0.1884 × attended post-secondary
+ 0.1173 × top income quintile
− 0 × (BMI < 23 & age < 45)
− 0.5520 × (23 ≤ BMI < 25 & age < 45)
− 0.9521 × (25 ≤ BMI < 30 & age < 45)
− 1.7162 × (30 ≤ BMI < 35 & age < 45)
− 2.3310 × (35 ≤ BMI & age < 45)
− 1.3602 × (BMI < 23 & age ≥ 45)
− 1.6537 × (23 ≤ BMI < 25 & age ≥ 45)
− 2.0563 × 25 ≤ (BMI < 30 & age ≥ 45)
− 2.5513 × (30 ≤ BMI < 35 & age ≥ 45)
− 2.9353 × (35 ≤ BMI & age ≥ 45).

Scale = 0.7994

Females:

μ = 10.5777
− 0.4098 × hypertension
− 0.4528 × non-white ethnicity
− 0.1477 × immigrant
+ 0.1939 × attended post-secondary
− 0 × (BMI < 23 & age < 45)
− 0.7432 × (23 ≤ BMI < 25 & age < 45)
− 1.1521 × (25 ≤ BMI < 30 & age < 45)
− 1.8479 × (30 ≤ BMI < 35 & age < 45)
− 2.0562 × (35 ≤ BMI & age < 45)
− 1.5832 × (BMI = missing & age < 45)
− 0.7100 × (BMI < 23 & 45 ≤ age < 65)
− 1.2338 × (23 ≤ BMI < 25 & 45 ≤ age < 65)
− 1.8357 × (25 ≤ BMI < 30 & 45 ≤ age < 65)
− 2.3742 × (30 ≤ BMI < 35 & 45 ≤ age < 65)
− 2.6631 × (35 ≤ BMI & 45 ≤ age < 65)
− 2.1988 × (BMI = missing & 45 ≤ age < 65)
− 1.5956 × (BMI < 23 & age ≥ 65)
− 1.6144 × (23 ≤ BMI < 25 & age ≥ 65)
− 1.9830 × (25 ≤ BMI < 30 & age ≥ 65)
− 2.2148 × (30 ≤ BMI < 35 & age ≥ 65)
− 2.6448 × (35 ≤ BMI & age ≥ 65)

− 2.4209 × (BMI = missing & age ≥ 65).

Scale = 0.8419

$$\mathbf{m} = \frac{\log(\text{follow-up time in days}) - \mu}{\text{scale}}$$

$$\mathbf{p} = 1 - \exp(-\exp^{m})$$

Number of diabetes cases = p * survey weights

References

1. Rosella LC, Lebenbaum M, Li Y, Wang J, Manuel DG. Risk distribution and its influence on the population targets for diabetes prevention. *Preventive medicine.* 2014;58:17-21.
